# Acupuncture Affects Autonomic and Endocrine but Not Behavioural Responses Induced by Startle in Horses

**DOI:** 10.1155/2015/219579

**Published:** 2015-08-30

**Authors:** Julia Dias Villas-Boas, Daniel Penteado Martins Dias, Pablo Ignacio Trigo, Norma Aparecida dos Santos Almeida, Fernando Queiroz de Almeida, Magda Alves de Medeiros

**Affiliations:** ^1^Department of Physiological Sciences, Federal Rural University of Rio de Janeiro, BR 465, KM 7, 23890 000 Seropédica, RJ, Brazil; ^2^Department of Physiology, School of Medicine of Ribeirão Preto, University of São Paulo, 14049-900 Ribeirão Preto, SP, Brazil; ^3^Veterinary Institute, Federal Rural University of Rio de Janeiro, BR 465, KM 7, 23890 000 Seropédica, RJ, Brazil; ^4^Multicenter Post-Graduation Program in Physiological Sciences, Brazilian Society of Physiology, 05508 000 São Paulo, SP, Brazil; ^5^Post-Graduation Program in Veterinary Medicine and Post-Graduation Program in Physiological Sciences, Federal Rural University of Rio de Janeiro, 23890 000 Seropédica, RJ, Brazil

## Abstract

Startle is a fast response elicited by sudden acoustic, tactile, or visual stimuli in a variety of animals and in humans. As the magnitude of startle response can be modulated by external and internal variables, it can be a useful tool to study reaction to stress. Our study evaluated whether acupuncture can change cardiac autonomic modulation (heart rate variability); and behavioural (reactivity) and endocrine (cortisol levels) parameters in response to startle. Brazilian Sport horses (*n* = 6) were subjected to a model of startle in which an umbrella was abruptly opened near the horse. Before startle, the horses were subjected to a 20-minute session of acupuncture in acupoints GV1, HT7, GV20, and BL52 (ACUP) and in nonpoints (NP) or left undisturbed (CTL). For analysis of the heart rate variability, ultrashort-term (64 s) heart rate series were interpolated (4 Hz) and divided into 256-point segments and the spectra integrated into low (LF; 0.01–0.07 Hz; index of sympathetic modulation) and high (HF; 0.07–0.50 Hz; index of parasympathetic modulation) frequency bands. Acupuncture (ACUP) changed the sympathovagal balance with a shift towards parasympathetic modulation, reducing the prompt startle-induced increase in LF/HF and reducing cortisol levels 30 min after startle. However, acupuncture elicited no changes in behavioural parameters.

## 1. Introduction

The startle response is a fast defensive mechanism that protects the organism against potential injury. The analysis of startle reactions can be a useful tool to assess stress and welfare, since the magnitude of startle can be modulated by chronic or acute manipulations [1–6]. For example, changes in emotional and perceptual homeostasis, that is, conditioned and unconditioned aversive events, can enhance the magnitude of acoustic startle response (ASR) [1, 3, 7]. Otherwise, a pleasant emotional context may lead to attenuated startle responses [4, 6, 8]. Although several kinds of stimuli (acoustic, tactile, or visual) can induce startle in animals and humans, the ASR is the most investigated one [9]. In horses, the exposure to a novel object (i.e., opening umbrella) has been used to produce startle response [10–12]. The startle model of opening umbrella can produce behavioural response (escape response), autonomic changes [increase in the heart rate (HR), increase in the power of the low frequency (LF) band of the cardiac interval spectrum and in the LF/HF ratio, and decrease in the power of the high frequency (HF) band], with no changes in the cortisol levels (unpublished data from our laboratory). Therefore, this model can be effectively used for neurological studies in horses.

Acupuncture has been used to reduce stress responses in humans and animals. The term acupuncture is derived from the Latin radicals “acus and pungere” meaning needle and puncture. This technique seeks to treat and cure diseases by applying stimuli through the skin, with the insertion of needles into specific points. In contemporary western medicine, acupuncture is considered a form of sensory stimulation that affects several classes of afferent nerve fibres and once directed to the spinal cord and the higher centres in the CNS, it triggers the neurophysiological events that generate the therapeutic outcome [13]. The stimuli generated by acupuncture can activate the autonomic centres and hypothalamic-pituitary-adrenal (HPA) axis and, thereby, help to maintain homeostasis and improve the efficiency of several systems of the organism [14]. Therefore, the stimulation of acupoints can prevent stress-induced changes in autonomic nervous system [14] and in HPA axis [15–17].

Although acupuncture has been used for treating various conditions in horses, the literature is scarce in studies concerning the effects of acupuncture in stress-related conditions in horses. In the present study we tested the hypothesis that a single session of acupuncture could lead to a sedation-like response prior to a startle test (abrupt opening of umbrella), changing behavioural response, cardiac interval variability, and cortisol levels in horses.

## 2. Methods

### 2.1. Animals

Six Brazilian Sport horses (3 males and 3 females; 6–8 years old; 450–550 kg of body mass), with appropriate body condition scores, obtained from the Brazilian Army Riding School were used in the experimental protocols. These horses had been undergoing, since they were 5 years old, a 6-day-week training routine including galloping, jumping, and dressage exercises. In the current study, the horses were housed in individual stalls and fed with concentrated cost-cross hay and had free access to tap water.

All experimental procedures were approved by the Committee on Animal and Human Research and Ethics of the Federal Rural University of Rio de Janeiro/COMEP-UFRRJ (protocol number 230833.002064/2012-10).

### 2.2. Experimental Design

Data collection was conducted on two consecutive days with experimental protocols starting at 06:00 am and ending around 10:00 am, in order to minimize circadian influences. Early in the morning a heart monitor (RS 800 G3, Polar, Kempele, Finland) was strapped to the chest of the horses to continuously record HR. After that the baseline blood samples (SB) were collected. The animals were then left to rest quietly for 20 minutes in their stalls (CTL; control group) or subjected to acupuncture in points or in nonpoints (detailed below). Next, each horse was taken individually to a covered arena (70 × 30 m, known by the animals and often used for dressage exercises) and subjected to the startle test, the abrupt opening of an umbrella [18]. Briefly, the horse was led by a known handler and positioned at a predetermined location, with its back to a low wall (70 cm high) that surrounds the arena, and held loosely by the halter. The horse was left undisturbed until signs of quietness and inattention were seen. Then, a rainbow coloured umbrella (70 cm total length) was suddenly opened and spun for 2 minutes by a person that was hidden behind the wall at a distance of approximately 1.5 m from the rump of the animal. The umbrella was positioned clearly in the visual field of the horse (an angle of approximately 45 degrees to the tail of the horse). Following the test, the horse was kept in the arena by the halter for an additional 3-minute period in order to videotape the behavioural responses. Finally, the horses were taken to their stalls and had blood samples collected at 30 (S30) and 60 (S60) minutes following the startle test.

### 2.3. Experimental Groups and Treatments

**Figure 1:**
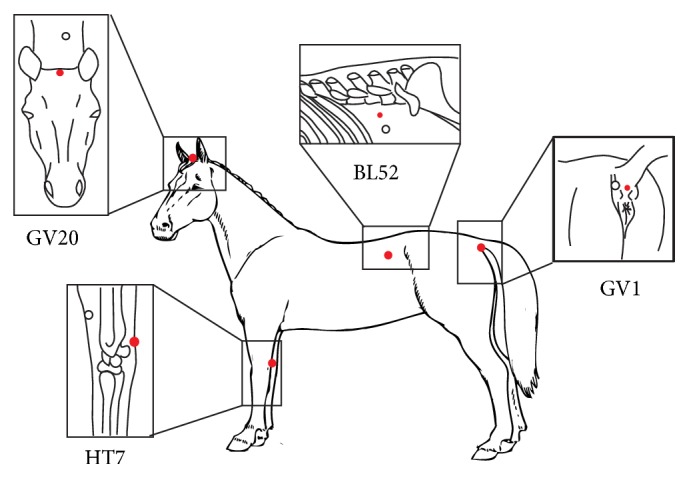
Outline of horse body showing the locations of the acupuncture points (red full circles) and nonpoints (open circles). BL: Bladder; GV: Governing Vessel; HT: Heart.

Horses were randomly assigned to three groups:

(i) *CTL*: control group: animals were kept for 20 minutes without any manipulation prior to startle test (*n* = 6);
(ii) *NP*: animals were subjected to insertion of needles at nonacupuncture points prior to startle test; sterile stainless steel needles 0.30 × 40 mm (Dongbang, Korea) were inserted and kept for 20 minutes to a depth of 1 cm at a location 3 to 5 cm from the acupuncture points GV1, HT7, GV20, and BL52 (described below), avoiding stimulation of other acupoints (*n* = 6) (Figure 1);
(iii) *ACUP*: animals were subjected to acupuncture prior to startle test; sterile stainless steel needles 0.30 × 40 mm (Dongbang, Korea) were inserted and kept for 20 minutes to a depth of 1 cm at the acupoints:* GV1* (Point 1 of Governing Vessel meridian,* Ho Hai* or* Chang Qiang*), located at a depression between the anus and the ventral tail base and innervated by rectal nerve flow;* HT7* (Point 7 of Heart meridian,* Shenmen*), located caudally to the radius bone, just proximal to the accessory carpal bone at the insertion of the carpal ulnar bone, and innervated by median and ulnar nerves;* BL52* (Point 52 of the Urinary Bladder meridian,* Zhi Shi*) located 6 cuns (1 cun = width of floating rib of the horse) lateral to the dorsal midline between the 2nd and 3rd spinous processes of lumbar vertebrae and innervated by dorsal cutaneous branch of the first lumbar nerve;* GV20* (Point 20 of the Governing Vessel meridian, Bai Hui), located at the highest point of the poll, rostral to the occipital crest, and innervated by auriculopalpebral nerve (*n* = 6) (Figure 1).

### 2.4. Cardiac Interval Variability Analysis

Cardiac intervals were continuously sampled using a heart monitor (RS 800 G3, Polar, Kempele, Finland). Following acquisition, the data were transmitted from the heart monitor to custom computer software (Polar Pro Trainer 5, Polar, Kempele, Finland) through an infrared interface. The recordings were then processed and the beat-by-beat time series of cardiac interval values were generated. Next, the time series were divided into data sets for the basal stall (horses in their stalls before the startle test), basal arena (horse in the arena, immediately before the test), startle, and after startle (horses in theirs stalls, 30 minutes after the startle test) periods.

The analysis of heart rate variability was performed using custom computer software (CardioSeries v2.4, http://www.danielpenteado.com) designed to perform time-frequency analysis of cardiovascular variability, allowing precise adjustment of the parameters related to this kind of analysis (e.g., interpolation rate, segment length, and boundaries of frequency bands). Beat-by-beat series of cardiac interval values were converted to evenly spaced data points using cubic spline interpolation (4 Hz). The interpolated series were divided into sequential sets of 256 data points (64 s) overlapped by 50%, which were detrended and tested for stationarity. The presence of slow trends in time series can affect the power of frequency bands of the spectra [19]. Before spectral calculation, time series were detrended by subtracting the linear trend (obtained by linear regression calculation) from data points [20].

It is worth mentioning that cardiovascular variability analysis requires at least weakly stationary data series (i.e., mean and stable covariance over time) [19, 21]. Data series stationarity can be tested by means of stationarity tests (i.e., enhanced reproducibility of the results among users and laboratories) [21, 22] and through visual inspection of data series [23–25]. In the current study, a well-experienced researcher visually inspected the segments of interpolated time series searching for transients that could affect power spectral density (PSD) calculation. To confirm that the visual inspection of the interpolated time series was properly conducted, a Hanning window was applied to attenuate side effects and the spectrum was calculated for all segments using a direct Fast Fourier Transform (FFT) algorithm for discrete time series. All segments were visually inspected for abnormal spectra. Lastly, the results from the inspections of the time series and spectra were taken together for the analysis and nonstationary data were not considered [26]. The spectra were integrated in the low frequency (LF; 0.01–0.07 Hz) and high frequency (HF; 0.07–0.50 Hz) bands [27]. The normalised values were achieved by calculating the percentage of LF and HF power with regard to the total power of the spectrum minus the very low frequency band (VLF; <0.01 Hz) power [24, 28]. To assess the sympathovagal balance, the LF/HF ratio of cardiac interval variability was calculated [27, 29–31].

### 2.5. Cortisol Analysis

Blood samples from the jugular vein were collected in SST Vacutainer tubes. Following the collection, the blood was centrifuged for 10 minutes at 3200 rpm. The serum (~3 mL) was collected in plastic tubes and kept at −20°C. Serum cortisol concentrations were determined, in duplicate, by a double antibody radioimmunoassay method using a commercial kit (RD Coated Tube Cortisol I125 RIA, Costa Mesa, CA, USA). The sensitivity of the assay was 0.17 *μ*g/dL and the intra-assay coefficient of variation was 6.59%.

### 2.6. Behavioural Analysis of Reactivity

The horses were videotaped by a camera (SDR H20, Panasonic, Tokyo, Japan) placed at a distance of 20 meters and the images were later processed and analysed by computer (ImageJ, U.S. National Institute of Health, http://rsb.info.nih.gov/nih-image). For the behavioural analysis, as previously shown in the literature [32], three parameters were assessed: (1)* latency of reaction*: time between the beginning of the test and the first reaction of the animal; (2)* duration*: total time spent in the motor response to the stimulus; and (3)* covered distance*: displacement of the animal in response to the stimulus.

### 2.7. Statistical Analysis

The cardiac interval variability and cortisol levels data were analysed by means of two-way analysis of variance (ANOVA) for repeated measures with the factors: group (CTL, NP, and ACUP) and time (HRV: basal stall, basal arena, startle, and after startle; and cortisol: SB, S30, and S60) and interaction group × time, followed by the multiple comparison Bonferroni test. Behavioural data were analysed using the one-way ANOVA. Differences were considered if *P* < 0.05. The results are shown as mean ± standard error of mean.

## 3. Results

**Figure 2:**
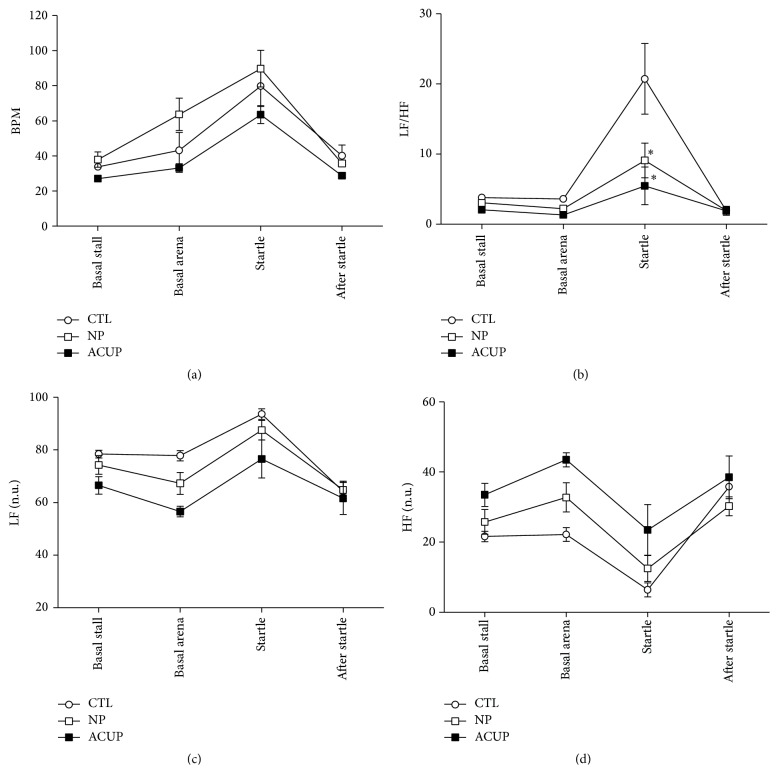
Effect of the acupuncture on the heart rate (HR, (a)), LF/HF ratio (b), and power of the low frequency (LF, (c)) and high frequency (HF, (d)) bands of the pulse interval spectrum of horses subjected to startle test. Data obtained from control horses (CTL; *n* = 5), horses subjected to 20-minute sessions of acupuncture at acupoints (ACUP; *n* = 4), and horses subjected to 20-minute sessions of acupuncture at nonpoints (NP; *n* = 4). ^*∗*^ *P* = 0.001 ACUP versus CTL at moment of startle.

In the analysis of HR, the two-way ANOVA for repeated measures detected differences at the factors group (*F*_(2,51)_ = 6.628; *P* = 0.015) and time (*F*_(3,51)_ = 25.611; *P* < 0.001), with no significance at interaction. The* Bonferroni Posttest* detected in the group factor that ACUP group had lower HR than NP group (*P* = 0.014), with no difference between the other groups. In the time factor, the moment startle also was higher than the other times (*P* < 0.001) (Figure 2(a)).

In the analysis of the LF/HF ratio, the two-way ANOVA for repeated measures detected differences at factors group (*F*_(2,51)_ = 6.003; *P* = 0.019), time (*F*_(3,51)_ = 16.929; *P* < 0.001), and interaction (*F*_(6,38)_ = 3.772; *P* = 0.006). The* Bonferroni Posttest* detected in the group factor that ACUP group had lower LF/HF than CTL group (*P* = 0.002), with no difference between the other groups. In the time factor, the ratio LF/HF was higher at the moment startle than at the other times (*P* < 0.001). In the interaction factor, only at the time startle the groups ACUP and NP were different from CTL (*P* < 0.001), with no difference between ACUP and NP (Figure 2(b)).

In the analysis of the power of the LF and HF bands, the two-way ANOVA for repeated measures detected differences at the factors group (LF: *F*_(2,51)_ = 6.335; *P* = 0.017; HF: *F*_(2,51)_  =  8.080; *P* = 0.008) and time (LF: *F*_(3,51)_ = 33,672; *P* < 0,001; HF: *F*_(3,51)_ = 25,919; *P* < 0,001), with no significance at interaction. The* Bonferroni Posttest* detected in the group factor that ACUP was different from CTL (LF: *P* = 0.016; HF: *P* = 0.008), with no difference between the other groups. In the time factor, the moment startle also was different when compared to the other times (LF and HF: *P* < 0.001) and the moment after startle was also different from the moment basal stall (LF: *P* = 0.002; HF: *P* = 0.029) (Figures 2(c) and 2(d)).

**Figure 3:**
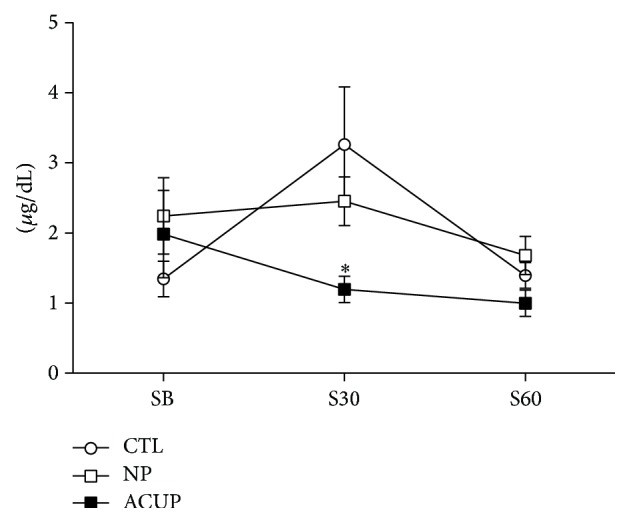
Effect of acupuncture on serum cortisol concentration of horses subjected to startle test. Data obtained from control horses (CTL; *n* = 6), horses subjected to 20-minute sessions of acupuncture at acupoints (ACUP; *n* = 6), and horses subjected to 20-minute sessions of acupuncture at nonpoints (NP; *n* = 5). Plasma was collected at baseline conditions SB (basal), S30 (after 30), and S60 (after 60) minutes of startle test. ^*∗*^ *P* < 0.01 versus CTL 30 minutes after startle.

In the analysis of serum cortisol, the two-way ANOVA for repeated measures detected differences at the time (*F*_(2,50)_  =  4.54; *P* = 0.02) and interaction factors (*F*_(4,51)_ = 3.18; *P* = 0.28), with no significance at the group factor. The* Bonferroni Posttest* detected that ACUP was significantly different from CTL at moment S30 (30 min after startle) (*P* < 0.01), with no difference between the other groups (Figure 3). This indicates that acupuncture reduces the startle-induced increase in serum cortisol (since the peak of cortisol was seen 30 min after stimulus).

In response to the umbrella opening, the horses of all studied groups showed a standard escape response, characterized by a small jump followed by a short gallop moving away from the opened umbrella. After this reaction, the animals remained immobile looking at the umbrella that was spun for 120 s after its opening. The horses exhibited little motion during the 5 minutes following startle test, since halters loosely contained them, but remained alert to the environment. The acupuncture did not affect the behavioural startle responses, since no difference was found between groups regarding latency (*P* = 0.1728), duration (*P* = 0.9754), and distance (*P* = 0.1845) (One-way ANOVA) (Table 1).

## 4. Discussion

Our results show that the startle test induced an increase in HR, in the power of the LF band of the cardiac interval spectrum, and in the LF/HF ratio, accompanied by a decrease in the power of the HF band of the cardiac interval spectrum, confirming the marked cardiac autonomic imbalance typically observed following startle stimulus [33, 34]. The stimulation in the acupoints GV1, HT7, GV20, and BL52 was able to shift the sympathovagal balance towards parasympathetic predominance. Looking at the HRV results, taking all time points together (basal stall, basal arena, startle, and after startle), when compared to control group the ACUP group exhibited reduced LF/HF ratio and LF band and increased HF band with no change in the HR, suggesting a noticeable effect of acupuncture on the sympathovagal balance. Furthermore, looking at only the time point startle, the acupuncture reduced markedly the startle-induced increase in the LF/HF ratio. As the values of LF and HF were not different among all groups studied, the decrease in the LF/HF ratio could be attributed to a simultaneous increase in the vagal tonus and decrease in the sympathetic tonus.

It is well known that acupuncture can regulate the autonomic function [14, 35]. Recently several studies have addressed the autonomic effect of stimulation of acupoints using the HRV analysis [14, 36, 37]. Although some studies have shown that the stimulation of some acupoints can increase the LF/HF ratio [38], reliable data have shown that acupuncture can reduce the LF/HF ratio, either by increasing the HF (high frequency) power or decreasing the LF (low frequency) power [37]. Acupuncture at* Sishencong* points, which are located on the vertex of the head, each 1 cm away from acupoint GV20 in four directions, increases HF and decreases LF power in healthy humans [39]. Saam acupuncture (gallbladder jeonggyeok), a traditional Korean acupuncture that focuses on the* Gallbladder* channel to control the spirit and sedate the mind, decreases the LF power and the LF/HF ratio and increases HF power in night-shift-working nurses [40]. It has also been reported that acupuncture applied at LI4 and PC6 in fatigued subjects induces a decrease in the LF power and the LF/HF ratio and an increase in the HF power [41]. In the view of the Traditional Chinese Medicine, acupuncture can restore the balance between “*Yin* and* Yang*” [42]. In the terminology of western medicine this can be translated as “acupuncture modulates the imbalance between the parasympathetic and sympathetic activity” [42]. Therefore, acupuncture has been proposed for the treatment of diseases related to the autonomic nervous system by modulating the balance between sympathetic and parasympathetic activities [14].

The effect of acupuncture on the HRV will mainly rely on the condition of the animal or subject (e.g., healthy, sick, or under stress) and the parameters of acupuncture (i.e., acupoints chosen and kind of stimulation) employed [43]. Many studies with positive effects of acupoints stimulation on the HRV status (i.e., reduction in the LF/HF ratio) have chosen combinations of acupuncture points to produce sedation and calming [40, 41]. Obviously the location of the acupoints and their effects on humans can differ from the acupoints used on horses. The literature about the specific effects of acupoints stimulation in horses is scarce. Thus, the acupoints used in this study were chosen based on the MTC's indication to treat emotional disorders in horses [44]. Also, the acupoints were chosen based on ease of accessibility, horse tolerance, and, when possible, previous data from the literature. The acupoint* GV20*, located at the highest point of the poll, rostral to the occipital crest, is stimulated to treat convulsions and ear and eye problems and promote tranquilization [44]. The* HT7*, located on the caudal aspect of radius, is also indicated to control anxiety and to induce mind quietness [44]. The* B52* acupoint is traditionally indicated for reproductive disorders, oedema, and constipation, but also it can be used to control fear [44]. The* GV1* acupoint is indicated for caudal back pain, constipation, and genitourinary problems and can calm the mind [44]. Pharmacopuncture with 1/10 of acepromazine doses at* GV1* produced a mild sedation (i.e., reduced excitement and reactivity to different stimuli), when compared with the conventional dose of acepromazine [45] in undisturbed (i.e., not subjected to stress) animals. In animals subjected to road transport the same stimulation reduced baseline HR and the transport-induced increase in HR at unloading, without changing respiratory rate, body temperature, and serum cortisol [46].

A recent study has shown that a single stimulation of acupoints* Bai Hui, PC6, SI9, BL54*,* and GV14* did not change the HRV parameters in healthy undisturbed horses [47]. The authors related this lack of effect of the acupuncture stimulation over HRV to some issues: (1) extremely high variance in HRV data; (2) chosen acupoints; (3) lack of ancillary stimulation; and (4) use of clinically normal horses. Since the study by Le Jeune and colleagues applied the HRV analysis using parameters comparable to the current study, we agree with those authors that the highlighted issues (e.g., HRV data variance and chosen acupoints) may have played a role in the acupuncture effects.

In the current study, it was found that acupuncture at nonacupoints elicited a reduction in the LF/HF ratio less pronounced than that observed in the group subjected to acupuncture at acupoints. This attenuated effect has already been shown in several studies [48–50], and it might be related to nonspecific effects of the needle insertion [51].

Our study also has shown that acupuncture reduces the startle-induced increase in serum cortisol. Previous studies have shown that acupuncture may reduce the stress-induced increase in cortisol [17, 52–54] and the mechanism of action of acupuncture has been associated with the blockade of the stress-induced HPA activation [15, 16, 55, 56]. Electroacupuncture (EA) at* HT3-PC6* acupoints significantly attenuated the immobilization-induced c-fos expression in the parvocellular portion of the Paraventricular Nucleus of Hypothalamus [55]. The EA at* St36* acupoint decreased the cold stress-induced elevation on the peripheral hormones [adrenocorticotropic hormone (ACTH) and cortisol], corticotropin-releasing hormone (CRH) levels, and adrenal neuropeptide Y (NPY) mRNA [16]. This mechanism can explain, at least in part, the acupuncture effect on the prevention of the deleterious effects of chronically high corticosteroids levels. Furthermore these results corroborate the fact that acupuncture can be used to treat stress-related disorders in horses.

In contrast to our results that did not show effects of the acupuncture on the startle-induced behavioural response, some studies showed that acupuncture may reduce the behavioural changes induced by stress [56–58]. In this respect, the peculiarities (type, duration, and repetition) of the stressor and the acupuncture treatment have to be considered. Studies in the literature show that more expressive results are seen when repetitive protocols of acupuncture are employed. Kim and colleagues (2009) stimulated repeatedly (daily for 4 weeks) the* PC6* acupoint to prevent the effects of a chronic mild stress (CMS) model on behaviours related to anxiety (i.e., elevated-plus maze test) and depression (i.e., sucrose intake test) [56]. The stimulation of the same acupoint (three times a week during 4 weeks) reverted the memory impairment (i.e., retention test) in the same model of stress [57] in rats. The stimulation of the* PC6* acupoint also attenuated CMS-induced Fos expression in the PVN [56] and increased AchE reactivity in the hippocampus [57]. In another model of chronic unpredictable mild stress (different protocol from that used by Kim and colleagues), the repetitive stimulation (alternate days for 4 weeks) at acupoints Baihui (Du20) and Neiguan (PC6) attenuated the behavioural deficit (open field and sucrose intake tests) and decreased the phosphorylated forms of extracellular signal-regulated kinase- (ERK-) cAMP response element binding protein (CREB) in the hippocampus and prefrontal cortex. The ERK-CREB signal pathway has been implicated in the pathogenesis of depression, suggesting that the antidepressant-like effect of acupuncture might be mediated by activating the ERK-CREB pathway in the brain [58].

Our study used a single stimulation with dry needles for 20 minutes (i.e., no ancillary stimulation such as pharmacopuncture or moxa) before the startle test. In fact, the clinical use of acupuncture usually includes 4 to 10 (or even more) acupoints given in each of the six (or even more) sessions. In general, studies that used protocols that resemble usual clinical practice are more likely to achieve better results [37]. This limitation could not be avoided in the current study but long-term acupuncture therapies should be employed in the next studies.

## 5. Conclusion

A single session of acupuncture at* GV1*,* HT7*,* GV20*, and* BL52* acupoints was able to attenuate the increase in cardiac sympathetic modulation and in cortisol levels induced by startle, without noticeable changes in the behavioural response. Our results suggest that acupuncture can modulate responses to startle in horses. In order to analyse the effect of acupuncture in different types of acute stress and mainly in the treatment of stress-related disorders in horses, future studies should consider different combination of acupoints and employment of long-term acupuncture therapies (i.e., over several days).

## Figures and Tables

**Table 1:** Behavioural responses to startle in horses.

	CTL (*n* = 6)	NP (*n* = 6)	ACUP (*n* = 6)	Value of *P*
Latency (s)	0.69 ± 0.41	0.45 ± 0.18	0.56 ± 0.14	0.1728
Duration (s)	4.46 ± 1.96	3.78 ± 0.79	3.72 ± 1.06	0.9754
Distance (m)	5.15 ± 2.75	3.08 ± 1.72	4.47 ± 1.24	0.1845

Latency: time until the animal reacted.

Duration: total time spent in the response.

Distance: displacement of the animal.
